# Vitamin D, C-Reactive Protein, and Cardiometabolic Risk Clustering in Middle-Aged Adults: Results from the 2023 Korea National Health and Nutrition Examination Survey (KNHANES)

**DOI:** 10.3390/biomedicines13112762

**Published:** 2025-11-12

**Authors:** Changhee Lee, Kyeongmin Jang

**Affiliations:** 1 Department of Paramedicine, Namseoul University, 91 Daehak-ro, Seonghwan-eup, Seobuk-gu, Cheonan-si 31020, Chungcheongnam-do, Republic of Korea; emtlch@naver.com; 2 Department of Nursing, College of Health Sciences, Daejin University, 1007 Hoguk-ro, Pocheon-si 11159, Gyeonggi-do, Republic of Korea

**Keywords:** metabolic syndrome, vitamin D, C-reactive protein, obesity, epidemiology, smoking, physical activity

## Abstract

**Background/Objectives**: Cardiometabolic risk clustering (CMRC), the coexistence of multiple risk factors, markedly increases the risk of cardiovascular disease and diabetes. While obesity is central, the independent roles of vitamin D status and systemic inflammation remain unclear. This study examined determinants of CMRC in middle-aged Korean adults, focusing on vitamin D and C-reactive protein (CRP). **Methods**: Cross-sectional data were analyzed from 2062 adults aged 40–64 years in the 2023 Korea National Health and Nutrition Examination Survey. CMRC was defined as ≥3 of abdominal obesity, hypertension, diabetes, hypertriglyceridemia, and low high-density lipoprotein cholesterol. Serum 25-hydroxyvitamin D [25(OH)D], CRP, lifestyle behaviors, and covariates were assessed. Complex-sample logistic regression identified factors associated with CMRC. **Results**: CMRC prevalence was 16.5%. Older age (OR = 1.04, 95% CI: 1.02–1.06), current smoking (OR = 1.76, 95% CI: 1.26–2.45), elevated CRP (1–3 mg/L: OR = 1.40, 95% CI: 1.04–1.87; ≥3 mg/L: OR = 1.63, 95% CI: 1.00–2.66), and obesity (OR = 8.29, 95% CI: 6.12–11.21) increased CMRC risk. Protective factors included male sex (OR = 0.60, 95% CI: 0.45–0.81), sufficient vitamin D (≥20 ng/mL: OR = 0.76, 95% CI: 0.58–0.99), and meeting World Health Organization physical activity guidelines (OR = 0.71, 95% CI: 0.55–0.92). **Conclusions**: These survey-weighted associations may help identify at-risk mid-life adults at the population level and motivate longitudinal evaluation of vitamin D deficiency and inflammation in risk assessment and targeted prevention.

## 1. Introduction

Cardiometabolic risk factors—including obesity, hypertension, dyslipidemia, impaired fasting glucose, and systemic inflammation—are recognized as major drivers of the global burden of chronic disease [1]. These risk factors frequently cluster within individuals, a phenomenon referred to as cardiometabolic risk clustering (CMRC) [2]. The simultaneous presence of multiple risk factors exerts additive and possibly synergistic effects, leading to substantially higher risks of cardiovascular disease, type 2 diabetes, and premature mortality compared with single risk exposures [3]. With population aging, CMRC has become an important focus for clinical management and public health policy [2].

In Korea, the prevalence of cardiometabolic conditions has risen markedly in recent decades, reflecting demographic shifts and lifestyle transitions [4]. Middle-aged adults (40–64 years) represent a critical stage in which cardiometabolic risks accumulate, yet preventive actions remain possible [5]. Identifying determinants of CMRC in this group is therefore essential for strategies that may delay or prevent disease progression.

Obesity is a well-established contributor to CMRC, primarily through links with insulin resistance, abnormal lipid metabolism, and hypertension [6]. However, exclusive attention to adiposity may overlook other modifiable influences. Increasing research has focused on vitamin D status as a potential correlate beyond adiposity. Vitamin D, mainly obtained through sunlight and diet, has traditionally been linked to bone and mineral metabolism [7], with emerging evidence for broader roles in insulin sensitivity, lipid regulation, and immune modulation [8,9]. Low serum 25-hydroxyvitamin D [25(OH)D] concentrations have been associated with insulin resistance, metabolic syndrome, and hypertension [10]; yet, evidence for clustering per se is heterogeneous once central adiposity and lipids are accounted for [11].

Systemic inflammation has likewise been implicated in CMRC. CRP, a marker of low-grade inflammation, has been linked to obesity, insulin resistance, vascular dysfunction, and atherosclerosis [12]. Elevated CRP levels are also associated with increased risks of metabolic syndrome and cardiovascular events independent of traditional risk factors [13]. However, the role of inflammation in CMRC among middle-aged adults, and its interaction with vitamin D or lifestyle factors, remains insufficiently examined.

Lifestyle behaviors—including smoking, alcohol use, physical activity, and sleep—further influence cardiometabolic health [14]. For example, adherence to the World Health Organization (WHO) physical activity guideline is protective, whereas smoking contributes to vascular dysfunction and insulin resistance [15]. Sleep duration, though less studied, has been linked to obesity and metabolic dysregulation [16]. Yet, the interplay between these behaviors, vitamin D status, and systemic inflammation in relation to CMRC is not fully clarified.

Taken together, current evidence highlights the multifactorial nature of CMRC while revealing knowledge gaps, particularly regarding the independent and combined roles of vitamin D [25(OH)D] and systemic inflammation (hs-CRP). Although Korea is a relevant setting—given rapid population aging and nationally representative health data—most prior studies examined these biomarkers in isolation, focused on older adults, or did not apply survey-weighted models to CMRC as an integrated outcome. Leveraging the most recent Korea National Health and Nutrition Examination Survey (KNHANES), our study addresses this gap by jointly evaluating 25(OH)D and hs-CRP in relation to CMRC in mid-life (40–64 years) using survey-weighted multivariable analyses.

We anticipated that lower serum 25(OH)D and higher hs-CRP would be associated with greater odds of CMRC in mid-life, independent of sociodemographic characteristics, lifestyle behaviors, and adiposity, and we explored their joint associations and potential sex differences. Using KNHANES 2023 and survey-weighted multivariable models, we aimed to (i) estimate the prevalence of CMRC in adults aged 40–64 years; (ii) quantify the independent and joint associations of 25(OH)D and hs-CRP with CMRC; and (iii) conduct prespecified sensitivity analyses (alternative 25(OH)D cut-offs; exclusion of extreme CRP values) and exploratory interaction checks to assess robustness.

## 2. Materials and Methods

### 2.1. Study Design and Data Source

This study employed a cross-sectional design using data from the 2023 KNHANES, which was released in December 2024. KNHANES is a nationally representative survey conducted annually by the Korea Disease Control and Prevention Agency (KDCA) to monitor the health and nutritional status of the Korean population. The survey consists of three components: a health interview, a health examination, and a nutrition survey. Participants are selected through a two-stage stratified cluster sampling design based on age, sex, and geographic region, ensuring representativeness of the civilian, non-institutionalized Korean population [17]. Detailed information regarding the survey design, sampling methods, and quality assurance procedures is available in the official KNHANES reports.

### 2.2. Study Population

**Figure 1:**
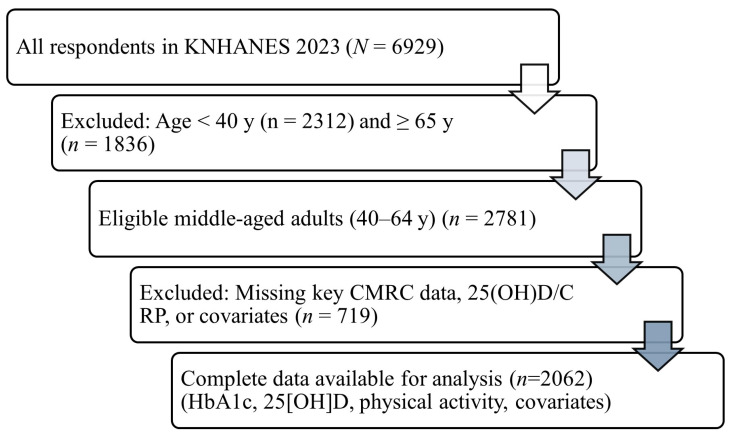
Flow of participant selection for the analytic sample, KNHANES 2023. From all respondents in 2023 (*n* = 6929), participants aged ≥65 years (*n* = 1836) and <40 years (*n* = 2312) were excluded to restrict analyses to middle-aged adults. Among the remaining 2781 participants, those with missing data for CMRC components, serum 25(OH)D, CRP, or prespecified covariates were excluded (*n* = 719), yielding a final analytic sample of *n* = 2062. Abbreviations: CMRC, cardiometabolic risk clustering; 25(OH)D, 25-hydroxyvitamin D; CRP, C-reactive protein; KNHANES, Korea National Health and Nutrition Examination Survey. Notes: CMRC was defined as the coexistence of ≥3 major metabolic risk factors (central adiposity, blood pressure/hypertension, dyslipidemia, impaired fasting glucose). Survey-weighted procedures (weights, strata, primary sampling units) were used in all analyses.

A total of 6929 individuals participated in the 2023 KNHANES. Of these, 1836 adults aged 65 years or older and 2312 individuals younger than 40 years were excluded to restrict the analysis to middle-aged adults. Among the remaining 2781 participants, 719 were further excluded due to missing data on study variables required for analysis. The final analytic sample, therefore, comprised 2062 adults aged 40–64 years with complete information on all variables of interest (Figure 1).

### 2.3. Measures

#### 2.3.1. Outcome Variable

CMRC was defined as the presence of three or more cardiometabolic risk factors, consistent with the criteria applied in a previous study [18]. The risk factors included abdominal obesity, hypertension, diabetes, hypertriglyceridemia, and low high-density lipoprotein (HDL) cholesterol. Abdominal obesity was defined as a waist circumference of ≥90 cm for men and ≥85 cm for women. Hypertension was defined as systolic blood pressure ≥ 130 mmHg, diastolic blood pressure ≥ 85 mmHg, or current use of antihypertensive medication. Diabetes was defined as fasting glucose ≥ 126 mg/dL, a physician diagnosis, or use of glucose-lowering medication. Hypertriglyceridemia was defined as triglycerides ≥ 150 mg/dL or current treatment for dyslipidemia, and low HDL cholesterol as <40 mg/dL for men and <50 mg/dL for women.

#### 2.3.2. Independent Variables

The primary independent variables of interest were serum 25(OH)D and high-sensitivity CRP. Serum 25(OH)D concentration was measured using liquid chromatography–tandem mass spectrometry, and vitamin D deficiency was defined as <20 ng/mL. Serum CRP concentration was measured by immunoturbidimetry and categorized into <1.0 mg/L, 1–3 mg/L, and ≥3 mg/L.

#### 2.3.3. Covariates

Sociodemographic covariates included age (continuous), sex (male/female), education (≤high school, ≥college), and household income (low–middle, middle–high). Lifestyle factors included alcohol use in the past year (yes/no), current smoking (yes/no), physical activity, and sleep duration. Physical activity was assessed according to the WHO guideline, classifying participants as meeting or not meeting the aerobic activity recommendation. Average sleep duration was dichotomized as <7 h/day vs. ≥7 h/day. Anthropometric and clinical measures included body mass index (BMI, kg/m^2^), waist circumference (cm), systolic and diastolic blood pressure (mmHg), fasting glucose (mg/dL), triglycerides (mg/dL), and HDL cholesterol (mg/dL).

Detailed diet quality, vitamin D intake/supplement use, season of blood draw, menopausal status, and medication use were unavailable/limited and could not be fully adjusted for.

### 2.4. Statistical Analysis

All models were estimated using IBM SPSS Complex Samples (v29) with sampling weights, strata, and primary sampling units (PSUs) to obtain design-based inferences. We prespecified covariates (age, sex, education, smoking, alcohol use, physical activity, adiposity, blood pressure/hypertension, lipids, and glycaemic markers/diabetes) while avoiding over-adjustment for components defining CMRC. Variance inflation factors (VIFs) were <2.5 in all multivariable models. Model performance included Nagelkerke R^2^; influence diagnostics were inspected. Sensitivity analyses varied 25(OH)D cut-offs (<10/<20/<30 ng/mL) and excluded extreme CRP values; vitamin D supplement use was not available and could not be adjusted for. We estimated survey-weighted logistic models (weights, strata, PSUs) and reported ORs (95% CIs). Multicollinearity was low (VIF < 2.5); model performance was summarized by Nagelkerke R^2^. Sensitivity varied 25(OH)D cut-offs and excluded extreme CRP values; exploratory interactions (sex × 25(OH)D, sex × CRP) were screened.

### 2.5. Ethical Considerations

The KNHANES is conducted by the KDCA under the National Health Promotion Act. All survey protocols were approved by the KDCA Institutional Review Board (IRB approval numbers: 2022-11-16-R-A). Written informed consent was obtained from all participants prior to data collection. De-identified datasets are publicly available for research purposes through the official KNHANES website. The present study was conducted in accordance with the ethical principles of the Declaration of Helsinki.

## 3. Results

### 3.1. Baseline Characteristics of the Study Population

A total of 2062 participants aged 40–64 years were included in the analysis. The mean age was 53.0 years (SD = 7.3), and 43.5% were male. Nearly half of the participants had attained at least a college education (46.4%), and 68.0% reported middle-to-high household income. The prevalence of current smoking and alcohol use in the past year was 19.1% and 77.0%, respectively. Approximately 46.4% of participants met the WHO physical activity guideline, and 55.7% reported sleeping ≥ 7 h per day. The mean body mass index (BMI) was 24.5 kg/m^2^ (SD = 3.6), and 40.0% were classified as obese (BMI ≥ 25 kg/m^2^). The mean serum 25(OH)D concentration was 24.4 ng/mL (SD = 10.8), while the mean CRP level was 1.29 mg/L (SD = 3.09). The average CMRC score was 1.25 (SD = 1.22) (Table 1).

### 3.2. Sociodemographic, Lifestyle, and Clinical Differences Between CMRC Groups

When stratified by CMRC status, significant differences were observed across multiple sociodemographic, lifestyle, and clinical characteristics (Table 2). Participants with CMRC were older (54.2 vs. 52.8 years, *p* = 0.001) and more likely to be male (64.5% vs. 39.1%, *p* < 0.001). Higher educational attainment (≥college) was less common in the CMRC group compared with the non-CMRC group (39.3% vs. 47.8%, *p* = 0.004), whereas household income did not differ significantly (*p* = 0.211). Regarding lifestyle behaviors, the CMRC group had higher rates of current smoking (29.0% vs. 17.1%, *p* < 0.001) and lower adherence to the WHO physical activity guideline (38.7% vs. 48.0%, *p* = 0.002). Average sleep duration was slightly shorter in the CMRC group (6.71 vs. 6.86 h/day, *p* = 0.018), although the proportion reporting ≥7 h of sleep per day did not differ significantly (*p* = 0.240). In terms of anthropometric and clinical markers, obesity was substantially more prevalent among participants with CMRC (80.1% vs. 32.0%, *p* < 0.001). The CMRC group also had higher BMI, waist circumference, systolic and diastolic blood pressure, fasting glucose, and triglyceride levels, alongside lower HDL-cholesterol and vitamin D levels (all *p* < 0.001). CRP levels were also significantly elevated in the CMRC group compared to the non-CMRC group (1.62 vs. 1.22 mg/L, *p* = 0.030).

### 3.3. Factors Independently Associated with CMRC

The results of multivariable logistic regression are presented in Table 3. Older age was independently associated with increased odds of CMRC (OR = 1.04, 95% CI [1.02–1.06], *p* < 0.001). Current smoking (OR = 1.76, 95% CI [1.26–2.45], *p* = 0.001), elevated CRP (1–3 mg/L: OR = 1.40, 95% CI [1.04–1.87], *p* = 0.026; ≥3 mg/L: OR = 1.63, 95% CI [1.00–2.66], *p* = 0.033), and obesity (OR = 8.29, 95% CI [6.12–11.21], *p* < 0.001) significantly increased the likelihood of CMRC. In contrast, male sex (OR = 0.60, 95% CI [0.45–0.81], *p* < 0.001), meeting the WHO physical activity guideline (OR = 0.71, 95% CI [0.55–0.92], *p* = 0.010), and sufficient vitamin D levels (≥20 ng/mL: OR = 0.76, 95% CI [0.58–0.99], *p* = 0.039) were protective factors. Education, household income, alcohol use, and sleep duration were not significantly associated with CMRC after adjustment for covariates.

## 4. Discussion

In this nationally representative sample of Korean adults aged 40–64 years, the prevalence of CMRC was 16.5%. In survey-weighted multivariable models, sufficient vitamin D (≥20 ng/mL) was associated with lower odds of CMRC, whereas elevated CRP (≥1.0 mg/L), obesity, and current smoking were associated with higher odds; meeting the WHO physical activity guideline was protective, while education, income, alcohol use, and sleep duration were not significant after adjustment. Sensitivity analyses varying vitamin D cut-offs and excluding extreme CRP values yielded directionally similar estimates (Supplement Tables S1 and S2). Taken together, these findings reinforce the multifactorial nature of CMRC, spanning biologic markers and modifiable behaviors, while we interpret them as associations given the cross-sectional design. Given the cross-sectional design, these patterns are associational and should be interpreted with caution.

### 4.1. Comparison with Previous Studies

The prevalence we observed is somewhat lower than in older populations where clustering is more common [19], consistent with evidence that cardiometabolic risk accumulates with age. Even so, a substantial proportion of mid-life adults already exhibit multiple risk factors, underscoring the need for targeted identification in this age group [20]. Our vitamin D finding aligns with reports of inverse associations between lower 25(OH)D and adverse metabolic profiles (insulin resistance, dyslipidemia, hypertension) [21], but evidence for clustering per se is heterogeneous once central adiposity and lipids are rigorously accounted for [13]. Such inconsistencies likely reflect differences in age structure, covariate adjustment (especially adiposity), and analytic approaches across studies [22].

The protective association of meeting the WHO physical activity guideline and the adverse association of current smoking are consistent with prior studies: physical activity improves insulin sensitivity, lipid metabolism, and blood pressure [23], whereas smoking contributes to inflammation and endothelial dysfunction [24]. Evidence linking sleep duration to metabolic dysregulation is less consistent [16].

Lifestyle patterns also played an important role. The protective effect of meeting the WHO physical activity guideline and the harmful effect of current smoking are consistent with prior studies. Physical activity improves insulin sensitivity, lipid metabolism, and blood pressure [23], while smoking contributes to inflammation and endothelial dysfunction [24]. The inverse association for male sex contrasts with reports of higher risk in men [25]. Differences in fat distribution, hormonal milieu, or behaviors may contribute, but these explanations are speculative. Where feasible, sex-stratified models and sex × 25(OH)D/sex × CRP interactions should be examined to clarify whether associations differ by sex. The inverse association for male sex contrasts with several reports; our exploratory interaction screens did not yield consistent evidence of effect modification. Formal sex-stratified analyses in longitudinal data are warranted.

### 4.2. Possible Mechanisms

Several biological and behavioral pathways may explain the observed associations. Vitamin D has been implicated in insulin secretion, lipid regulation, and blood pressure control through effects on pancreatic β-cell function, insulin receptor activity, the renin–angiotensin–aldosterone system, and vascular endothelial health [26]. Deficiency may therefore be compatible with a pro-atherogenic and pro-inflammatory milieu that co-occurs with CMRC, although causal pathways remain to be established [10].

Systemic inflammation, as indicated by elevated CRP, may also play a central role. CRP, produced in response to cytokines such as interleukin-6 and tumor necrosis factor-α, reflects chronic low-grade inflammation that promotes insulin resistance, endothelial dysfunction, and dyslipidemia [27]. Elevated CRP may thus serve as both a marker and mediator of cardiometabolic dysregulation [28]. Part of the CRP–CMRC association may be mediated or confounded by adiposity-related pathways, consistent with reports of attenuation after adiposity adjustment [29].

Lifestyle behaviors further modulate these pathways. Physical activity reduces inflammation and improves insulin sensitivity and body composition, while smoking induces oxidative stress and vascular injury, exacerbating metabolic dysfunction [23,24]. These mechanisms align with our findings that adherence to the WHO physical activity guideline was protective, whereas current smoking increased the risk of CMRC.

Finally, the observed sex differences may reflect hormonal and physiological distinctions. Estrogen exerts protective effects on lipid metabolism and vascular function, which decline after menopause, while differences in fat distribution between men and women may also contribute [30]. However, the precise mechanisms underlying these sex-specific associations remain to be clarified.

### 4.3. Public Health and Clinical Implications

The findings of this study have important implications for public health and clinical practice. Vitamin D sufficiency was associated with lower odds of CMRC, suggesting that improving vitamin D status may help reduce cardiometabolic risk. Although causality cannot be inferred, intervention studies indicate that supplementation can improve insulin sensitivity, glucose metabolism, and lipid profiles in deficient individuals [31]. Public health measures such as fortification, supplementation, and safe sun exposure could therefore be beneficial in Korea.

The independent association between elevated CRP and CMRC highlights the role of inflammation in cardiometabolic health. Routine CRP measurement, a simple and widely available test, may provide added value in identifying individuals at increased risk of clustering [32]. Incorporating inflammatory markers into preventive strategies could be especially useful for middle-aged adults not yet meeting clinical thresholds for disease. The protective effects of physical activity and the risks of smoking reaffirm the importance of lifestyle interventions. Promoting exercise and supporting smoking cessation remain priorities for chronic disease prevention, and their benefits appear to extend beyond single outcomes to CMRC more broadly [23]. These benefits, well established for single outcomes, appear to extend to CMRC at the population level, consistent with our associational findings.

Finally, the higher risk observed among women suggests that middle-aged Korean women may represent a particularly vulnerable subgroup. This underscores the need for sex-specific approaches to risk assessment and prevention, and for further research into the biological and social determinants of these patterns.

### 4.4. Strengths and Limitations

This study has several strengths. We used data from the 2023 KNHANES, a nationally representative survey with standardized measurements and complex sampling. Analyses were conducted with survey-weighted models to obtain design-based inferences. The sample of middle-aged adults afforded adequate precision to examine associations across sociodemographic, lifestyle, and clinical domains. In addition, we concurrently evaluated biochemical markers (vitamin D and CRP) and behavioral factors (smoking, physical activity, and sleep), enabling a comprehensive assessment of CMRC. Model diagnostics indicated low multicollinearity (VIF < 2.5) and modest explanatory power (Nagelkerke R^2^ ≈ 0.27), typical of multifactorial population models. Prespecified sensitivity analyses (alternative vitamin D cut-offs; exclusion of extreme CRP values) yielded directionally similar results, supporting robustness.

Several limitations warrant emphasis. First, the cross-sectional design precludes causal inference because temporality cannot be established. Second, vitamin D supplement use and detailed dietary information were unavailable, and information on the season of blood draw was limited, which may introduce residual confounding. Third, lifestyle variables (alcohol use, smoking, sleep duration) were self-reported and thus susceptible to recall or misclassification. Fourth, additional unmeasured factors—such as menopausal status and medication use (e.g., statins, antihypertensives)—could not be fully addressed. Fifth, single-time-point measurements of 25(OH)D and CRP may not capture long-term status or variability; however, our findings were robust to the exclusion of extreme CRP values in sensitivity analyses.

Although estimates are derived from a Korean national sample, the biological links among vitamin D, adiposity, lipids, and glycemic regulation are broadly relevant across populations. Even so, external validity beyond East Asia requires confirmation. We therefore interpret our results as associational and hypothesis-generating, and encourage multicountry replication using harmonized CMRC definitions and longitudinal designs.

## 5. Conclusions

In this nationally representative sample of Korean adults aged 40–64 years, approximately one in six exhibited CMRC. In survey-weighted models, sufficient vitamin D and meeting World Health Organization physical activity guidelines were associated with lower odds of CMRC, whereas elevated hs-CRP, obesity, and current smoking were associated with higher odds. These findings may inform risk identification at the population level but should be interpreted as associations rather than causal effects given the cross-sectional design. Longitudinal and interventional studies—incorporating detailed diet, seasonality, medication, and sex-specific analyses—are warranted to confirm these associations and to evaluate their utility in risk assessment and targeted prevention for mid-life populations.

## Figures and Tables

**Table 1:** Descriptive statistics of participant characteristics (N = 2062).

Variable	Category	n (%) or M (SD)
Age (years)	—	53.04 (7.34), range 40–64
Sex	Male	896 (43.5)
Education	≥College	957 (46.4)
Household income	≥Middle–high	1402 (68.0)
Alcohol use (past year)	Yes	1587 (77.0)
Current smoking (cigarette)	Yes	393 (19.1)
WHO PA guideline met	Yes	957 (46.4)
Sleep duration	≥7 h/day	1148 (55.7)
Obesity	≥BMI 25 kg/m^2^	824 (40.0)
CRP (mg/L)	—	1.29 (3.09), range 0.2–53.0
Vitamin D (25(OH)D, ng/mL)	—	24.35 (10.82), range 3.71–102.05
BMI (kg/m^2^)	—	24.54 (3.62), range 15.5–40.6
CMRC score (0–5)	—	1.25 (1.22)
	0	716 (34.7)
	1	581 (28.2)
	2	424 (20.6)
	3	231 (11.2)
	4	93 (4.5)
	5	17 (0.8)

Note: Values are presented as mean (standard deviation, SD) for continuous variables and number (percentage) for categorical variables. CRP = C-reactive protein; BMI = body mass index; PA = physical activity; WHO PA guideline = World Health Organization physical activity guideline.

**Table 2:** Comparison of participant characteristics according to CMRC (N = 2062).

Variable	CMRC Absent (n = 1721) n (%) or M (SD)	CMRC Present (n = 341) n (%) or M (SD)	χ^2^/t	*p*
Sociodemographic characteristics				
Age (years)	52.81 (7.36)	54.20 (7.14)	−3.20	0.001
Male	672 (39.1)	220 (64.5)	39.90	<0.001
Education ≥ college	823 (47.8)	134 (39.3)	8.32	0.004
Household income (middle–high)	1180 (68.6)	222 (65.1)	1.57	0.211
Lifestyle factors				
Alcohol use (past year, yes)	1329 (77.2)	258 (75.7)	0.39	0.531
Current smoker (cigarette)	294 (17.1)	99 (29.0)	26.34	<0.001
WHO PA guideline met (yes)	825 (48.0)	132 (38.7)	9.74	0.002
Average sleep duration (h/day)	6.86 (1.14)	6.71 (1.19)	2.37	0.018
Sleep ≥ 7 h/day	968 (56.2)	180 (52.8)	1.38	0.240
Anthropometric measures				
Obese (BMI ≥ 25 kg/m^2^)	551 (32.0)	273 (80.1)	273.80	<0.001
BMI (kg/m^2^)	23.90 (3.35)	27.79 (3.59)	−19.76	<0.001
Waist circumference (cm)	83.11 (9.4)	90.52 (8.6)	−14.29	<0.001
Clinical and biochemical markers				
Systolic BP (mmHg)	117.52 (14.6)	123.18 (12.9)	−7.24	<0.001
Diastolic BP (mmHg)	75.69 (9.5)	77.82 (8.9)	−4.40	<0.001
Fasting glucose (mg/dL)	98.84 (20.9)	117.97 (35.6)	−15.20	<0.001
HDL-cholesterol (mg/dL)	50.59 (14.7)	42.75 (13.5)	9.92	<0.001
Triglycerides (mg/dL)	118.97 (83.5)	231.77 (158.3)	−12.30	<0.001
CRP (mg/L)	1.22 (3.05)	1.62 (3.28)	−2.07	0.030
Vitamin D (25(OH)D, ng/mL)	24.08 (7.37)	22.61 (7.13)	3.63	<0.001

Note: Values are presented as mean (standard deviation, SD) for continuous variables and number (percentage) for categorical variables. Group differences were tested using χ^2^ tests for categorical variables and independent *t*-tests for continuous variables. CMRC = cardiometabolic risk clustering; CRP = C-reactive protein; BMI = body mass index; BP = blood pressure; PA = physical activity; HDL = high-density lipoprotein. No formal multiple-comparison adjustment was applied; *p*-values are descriptive, and effect sizes/precision are emphasized.

**Table 3:** Survey-weighted multivariable logistic regression: associations of 25(OH)D, hs-CRP, and covariates with CMRC (N = 2062).

Predictor	B	*p*	aOR	95% CI for OR
Age (years)	0.034	<0.001	1.035	1.015–1.055
Male sex	−0.505	<0.001	0.603	0.449–0.810
Education (≥middle school)	−0.202	0.166	0.817	0.617–1.084
Household income (middle-high)	−0.025	0.860	0.975	0.734–1.296
Current smoking	0.562	0.001	1.755	1.255–2.453
Alcohol use (past year)	−0.227	0.158	0.797	0.582–1.091
WHO PA guideline met (yes)	−0.344	0.010	0.709	0.545–0.923
Sleep ≥ 7 h/day	−0.033	0.801	0.967	0.748–1.251
CRP 1–3 mg/L	0.334	0.026	1.396	1.041–1.873
CRP ≥ 3 mg/L	0.490	0.033	1.633	1.003–2.658
Vitamin D ≥ 20 ng/mL	−0.278	0.039	0.757	0.581–0.987
Obesity (BMI ≥ 25 kg/m^2^)	2.115	<0.001	8.286	6.123–11.212

Note: Odds ratios (ORs) and 95% confidence intervals (CIs) were estimated using survey weights, strata, and primary sampling units (PSUs). The multivariable model adjusted for age (years), sex (Ref. = Female), education (≤high school/≥college), household income (quartiles), smoking (current vs. non-current), alcohol use (yes/no or risk category), physical activity (meets WHO guideline vs. not), and sleep duration (hours); waist circumference (cm) was included to account for adiposity. All VIFs < 2.5; Nagelkerke R^2^ is reported.

## Data Availability

The data that support the findings of this study are openly available from the KNHANES website (https://knhanes.kdca.go.kr, accessed on 12 July 2025), managed by the Korea Disease Control and Prevention Agency.

## Supplementary Materials

The following supporting information can be downloaded at: https://www.mdpi.com/article/10.3390/biomedicines13112762/s1, Table S1: Survey-weighted logistic regression for cardiometabolic risk clustering using alternative 25(OH)D cut-offs; Table S2: Survey-weighted logistic regression for cardiometabolic risk clustering after excluding participants with extreme CRP values.

## Abbreviations

The following abbreviations are used in this manuscript:

CMRC	Cardiometabolic Risk Clustering
CRP	C-Reactive Protein
WHO	World Health Organization
KNHANES	Korea National Health and Nutrition Examination Survey
KDCA	Korea Disease Control and Prevention Agency
IRB	Institutional Review Board
BMI	Body Mass Index
HDL	High-Density Lipoprotein
SD	Standard Deviation
OR	Odds Ratio
CI	Confidence Interval
25(OH)D	25-Hydroxy Vitamin D
