# Supplementary material for: Vitamin D, C-Reactive Protein, and Cardiometabolic Risk Clustering in Middle-Aged Adults: Results from the 2023 Korea National Health and Nutrition Examination Survey (KNHANES)
Source: Biomedicines. 2025 Nov 12;13(11):2762. doi: 10.3390/biomedicines13112762 (PMC12650019; doi:10.3390/biomedicines13112762)
Supplement: Supplementary file 1 [file biomedicines-13-02762-s001.zip › biomedicines-3881850-supplementary.pdf]

Supplementary Table S1. Survey-weighted logistic regression for CMRC using alternative 25(OH)D cut-offs

25(OH)D cut-off (deficiency)	Predictor (reference)	aOR	*p*	95% CI
<20 ng/mL (Base model)	25(OH)D ≥20 ng/mL vs <20	0.757	.039	0.581–0.987
<10 ng/mL	25(OH)D ≥10 ng/mL vs <10	0.808	.109	0.62–1.05
<30 ng/mL	25(OH)D ≥30 ng/mL vs <30	0.916	.470	0.74–1.15

Note. All models were fitted with survey weights, strata, and PSUs (SPSS Complex Samples logistic regression). Covariates matched the main model: age, sex, education, household income, current smoking, alcohol use in the past year, meeting WHO physical-activity guideline, sleep ≥7 h/day, CRP category, and obesity (BMI ≥25 kg/m^2).
CMRC = cardiometabolic risk clustering; 25(OH)D = 25-hydroxyvitamin D; aOR = adjusted odds ratio; CI = confidence interval.

Supplementary Table S2. Survey-weighted logistic regression for CMRC after excluding extreme CRP values

Analysis set	Exclusion rule	Predictor (reference)	aOR	*p*	95% CI
Base	—	25(OH)D ≥20 ng/mL vs <20	0.757	.039	0.581–0.987
Extreme-CRP excluded	CRP >10 mg/L	25(OH)D ≥20 ng/mL vs <20	0.740	.025	0.57–0.96

Note. Sensitivity analysis excludes likely acute-inflammation cases (CRP >10 mg/L). Covariates as in the main model.
CMRC = cardiometabolic risk clustering; CRP = C-reactive protein; aOR = adjusted odds ratio; CI = confidence interval; 25(OH)D = 25-hydroxyvitamin D.
